# Recent Advancements in Novel Sensing Systems through Nanoarchitectonics

**DOI:** 10.3390/bios13020286

**Published:** 2023-02-16

**Authors:** Karthick Velu, Rekha Goswami Shrestha, Lok Kumar Shrestha, Katsuhiko Ariga

**Affiliations:** 1 International Center for Materials Nanoarchitectonics (WPI-MANA), National Institute for Materials Science (NIMS), 1-1 Namiki, Tsukuba 305-0044, Japan; 2 Centre for Ocean Research, Sathyabama Institute of Science and Technology, Jeppiaar Nagar, Rajiv Gandhi Salai, Chennai 600119, India; 3 Department of Materials Science, Faculty of Pure and Applied Sciences, University of Tsukuba, 1-1-1 Tennodai, Tsukuba 305-8573, Japan; 4 Department of Advanced Materials Science, Graduate School of Frontier Sciences, The University of Tokyo, 5-1-5 Kashiwanoha, Kashiwa 277-8561, Japan

**Keywords:** nanoarchitectonics, VOCs, atomic/molecular manipulation

## Abstract

The fabrication of various sensing devices and the ability to harmonize materials for a higher degree of organization is essential for effective sensing systems. Materials with hierarchically micro- and mesopore structures can enhance the sensitivity of sensors. Nanoarchitectonics allows for atomic/molecular level manipulations that create a higher area-to-volume ratio in nanoscale hierarchical structures for use in ideal sensing applications. Nanoarchitectonics also provides ample opportunities to fabricate materials by tuning pore size, increasing surface area, trapping molecules via host–guest interactions, and other mechanisms. Material characteristics and shape significantly enhance sensing capabilities via intramolecular interactions, molecular recognition, and localized surface plasmon resonance (LSPR). This review highlights the latest advancements in nanoarchitectonics approaches to tailor materials for various sensing applications, including biological micro/macro molecules, volatile organic compounds (VOC), microscopic recognition, and the selective discrimination of microparticles. Furthermore, different sensing devices that utilize the nanoarchitectonics concept to achieve atomic-molecular level discrimination are also discussed.

## 1. Introduction

Fabricating materials through atomic/molecular manipulation, chemical harmonization, tuning to recognize certain functional groups, and spatial design confinements to trap specific molecules based on chemical heterogeneity are techniques that are used to delineate sensors via nanoarchitectonics [1,2]. Interaction of materials at the nanoscale level depends on atomic/molecular level changes that are observed as fluctuations at the quantum level, and vary with interactions at solid, liquid, or air–water interfaces [3,4]. Various non-covalent interactions through supramolecular chemistry, including van der Waals interactions, self-assembly, the Langmuir–Blodgett technique, layer-by-layer assembly, and electrostatic interactions, are used to create unique physical and chemical properties that are suitable for sensing a specific molecule [5,6]. Self-assembled structures for perfect order, such as stimuli responsiveness, hierarchic structures, cross-linking, and degree of porosity, can be useful for such applications [7,8]. Porous materials that are designed via nanoarchitectonics can enable the free flow of ions through a narrow channel, creating a diffusion path and place for confined molecules to interact (i.e., diffusion of solvent/gas molecules) [9,10]. Nanoscale modifications depend on the molecule’s energy and state, including internal dynamics, rotational, vibrational, electronic, and spin states [11,12]. The growth of nanoscale materials at interfaces by weakly interacting layer-by-layer (LbL) assemblies can offer tremendous control over structures, making them suitable for sensing applications through molecular recognition [13,14]. Materials that are structurally transformed via nanoarchitectonics can enhance atomic/molecular level discrimination of solvent vapors, biomolecules, and diagnostic applications [15,16]. Intramolecular vibrations and spatial confinement can make nanoporous/mesoporous platforms effective sensing systems for cooperative adsorption [17,18].

Soft nanoarchitectonics involve delicate processes that can produce highly ordered structures with extraordinary properties [19]. The ability to discriminate specific molecules by manipulating the shape of nanostructures is critical for developing efficient sensing platforms [20,21]. A sensing material’s hallmark is recognizing solvent vapors with similar molecular weights and different properties. Several sensing platforms have been developed, including tunable nanoresonators consisting of free-standing 3D nanoarchitectures for an enhanced Young’s modulus, have been developed [22,23]. Hierarchical nanostructured materials incorporated into nanomechanical sensors can elicit analyte-induced stimuli with nanometer precision [24,25]. Nanoporous materials create prolonged diffusion of guest molecules, which interact with the active sites via host–guest interactions to increase reliability [26,27]. One of the promising methods for detecting biomolecules is the covalent attachment of fluorophores with oligodeoxynucleotide, through fluorescence resonance energy transfer (FRET)-based detection [28,29]. Metal nanomaterials-based sensing platforms reflect changes via spatial proximity, resulting from a mismatch between the size of target molecules, and the decay length of localized surface plasmon resonance (LSPR) signals [30,31]. Sensing platforms that utilize the LSPR capability are used to detect biomolecules in clinical practice. In particular, noble metal nanomaterials with unique shapes and tips can generate hotspots for sensing biomolecules [32,33]. Electromagnetic enhancement, combined with plasmon excitation in metal nanomaterials, serves as a surface-enhanced Raman scattering (SERS) substrate for the detection of molecules that transfer electrons and form a metal–biomolecule interaction [34,35]. Fullerene-based sensing systems are generally regarded as highly selective to aromatic vapors through various mechanisms, such as strong π−π interactions, van der Waals interactions, bonding interplay, and so on [36,37]. Post-modification of fullerene assemblies can have a significant role in improving the sensitivity towards solvent vapor sensing. Chemical etching and amination reactions can be used to create numerous reactive sites (i.e., pores/nanostructures) for sensing applications [38,39]. This review focuses on recently developed sensor systems that apply the nanoarchitectonics concept, including devices and sensor materials. From the viewpoint of nanoarchitectonics, (a) tuning/manipulating shapes for creating hierarchical structures and (b) atomic/molecular fabrications for improving the effectiveness of sensing platforms will be discussed.

## 2. Sensing Biomolecules via Nanoarchitectonics

The detection of small biomolecules in in vivo systems can be significant for determining the severity of acute diseases, which are often less efficient in medical tests [40,41]. Functional nucleic acid-based sensors gain interest in detecting biomolecules and changes in the chemical and biological environment. Particularly, DNA aptamers, which consist of single-stranded DNA with specific base pairs, are often crucial in detecting metabolites, therapeutic molecules, and so on [42,43]. Aptamer-based sensors are widely used to detect circulating tumor cells via fluorophore labeling, pH responsiveness, and so on [44,45]. In some cases, nucleic acids were employed for the detection of biomolecules through DNA nanoarchitectonics [46,47]. In particular, DNA walkers, made of DNA origami for detecting single-nucleotide polymorphisms, interactions with other biomolecules, mutations, and structural alterations, are considered to be prospective biosensors [48,49]. Nucleic acids are promising materials, with unique electron transfer capacities and chemical heterogeneity [50,51]. Fluorescent dye is usually tagged to the oligodeoxynucleotide to enhance the SERS signal, which alters the conformation of the nucleic acid with respect to pH changes [52,53]. Very recently, our group has developed DNA-based solid thin films for the detection of solvent vapors, using DNA from salmon sperm. The laser molecular beam deposition technique was used to fabricate a flat sensing system that can effectively detect methanol from other solvent vapors [54]. Sun and co-workers have developed an aptamer-based sensing system conjugated with a gold surface to form a nanotetrahedron scaffold to specifically recognize human hepatocellular carcinoma tumor cells [55]. Microcystin-leucine arginine is a toxin that is produced by cyanobacteria, and is present in water for human consumption [56]. The detection of microcystin-leucine arginine is often challenging, as it is present in trace quantities. A recent study found that single-walled carbon nanohorn-based electrochemical immunosensors showed enhanced detection abilities. The presence of carboxylic groups around the tips of carbon nanohorns, and their interactions with the amino group of microcystin-leucine arginine, are critical for the efficiency of the sensing system [57]. Likewise, Pang and collaborators developed an enzyme-free electrochemical immunosensor system for detecting microcystin-leucine arginine, using molybdenum disulfide nanosheet/bovine serum albumin-stabilized gold nanocluster composite and gold-core platinum-shell nanoparticles [58]. Cucurbit[n]urils are a class of novel compounds, which derive their name from pumpkin’s “Cucurbitaceae”, and are known for their unique properties, such as hydrophobic inner cavities and fringed carbonyl portals. Molecules such as thiamine can be detected accurately using cucurbit[7]uril-based sensors via host–guest chemistry from oxidized thiamine (thiochrome) [59,60]. Likewise, cucurbit[6]uril-based hierarchic assemblies are used to detect nitroaromatic compounds in explosives. They make structural changes upon interacting with the nitroaromatic compounds, and produce a fluorescence-quenching effect that is used for detection [61]. Noncovalent interactions between atoms/bonds can be beneficial for the recognition of specific molecules, ranging from biomolecules to VOCs. Guanine-rich sensors utilize non-covalent π-stacking interactions that involve nucleobases for strong sensing of nucleic acid inhibitors [62]. Chen and collaborators developed an α-hemolysin-based nanopore stochastic sensing platform to recognize anions, cations, and hydrophobic molecules [63]. Similarly, Xiang and co-workers developed carbon nanotube/polyurethane-based flexible piezoresistive strain sensors. They used 1-pyrenecarboxylic acid to enhance the interface between carbon nanotubes and polyurethane, via non-covalent interactions [64].

**Figure 1:**
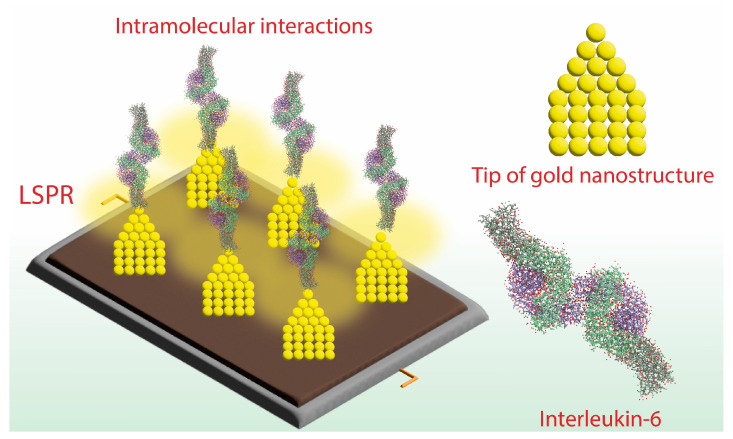
Tip-of-metal nanostructures generate strong LSPR for intramolecular interaction with biomolecules.

Collective oscillation of free electrons resulting in LSPR, and its biomolecular interactions, cause a change in the refractive index of the sensing surface. Tunable plasmonic nanostructures are ideal for sensing biologicals such as antigens, antibodies, and other sensitive molecules [65,66]. Often, these nanosystems use plasmonic materials such as gold, silver nanomaterials, and so on [67,68]. Luo and co-workers recently developed a cauliflower-like noble metal nanoparticle-based sensing system that utilized the LSPR effect. The developed system was tested for its sensing capability to detect interleukin-6 by measuring changes in the refractive index. The system showed a shift in the plasmon band in the thin film instead of bulk gold, indicating its sensitivity in detecting small biomolecules. The authors believed that the sensing mechanism could be due to the generation of hotspots by dense nanostructures with concavo-convex structures, which improves sensitivity through the enhancement of localized electromagnetic fields [69] (Figure 1).

Chang and collaborators fabricated a metal-insulator-metal nanodisk, in combination with polydimethylsiloxane, for the detection of cancer cells [70]. In another study, human angiotensin-converting enzyme 2 protein functionalized silver nanoparticles system showed rapid detection of severe acute respiratory syndrome coronavirus 2 through a plasmonic effect of the silver nanoparticles [71]. Qiu and co-workers developed gold nanoislands functionalized with poly(m-phenylenediamine-co-aniline-2-sulfonic acid) for the detection of lead cations in drinking water [72]. The researchers developed a boron-affinity magnetic immune SERS sensor for the detection of alpha-fetoprotein [73]. The 4-mercaptophenylboronic acid-modified metal nanoparticles were grafted and used as a detection probe. They used alpha-fetoprotein antibodies for successful tagging onto boric acid-functionalized magnetic silica particles, and used them as a magnetic immunocapture probe [74]. The developed system showed excellent sensitivity in detecting alpha-fetoprotein in both serum and clinical samples. The authors believed that the synergistic effect between “hotspots” caused by magnetic induction and the Raman peak intensity of each “hotspot” could be the reason for the sensitivity [75,76]. The sensing system showed enhanced specificity between antibodies of human liver carboxylesterase-1, Immunoglobulin G, bovine serum albumin, human serum albumin, and lipoprotein lipase [73,77].

**Figure 2:**
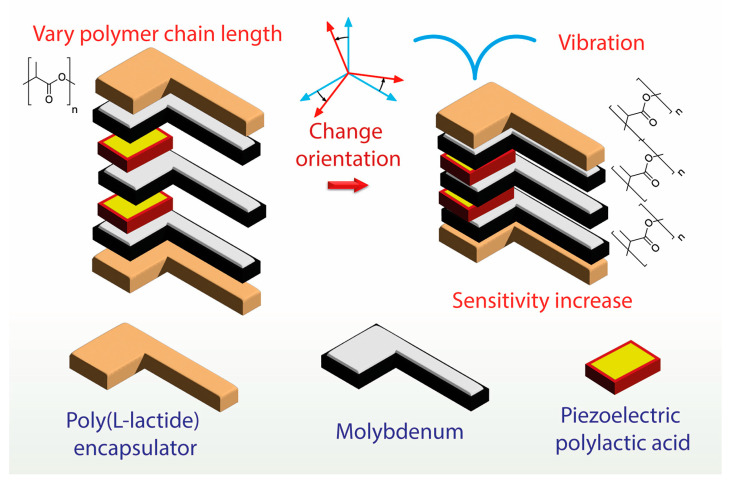
Biodegradable piezoelectric Poly(L-lactide) (PLLA) sensor to monitor pressure of diaphragmatic contraction in animal subjects.

The molybdenum disulfide nanosheet/bovine serum albumin-stabilized gold nanocluster composite showed greater surface area for reactivity and excellent biocompatibility. Researchers used gold-core platinum-shell nanoparticles as an enzymatic reporter to recognize microcystin-leucine arginine antibodies [58]. Silicon nanowire-based field effect transistor sensors with a permeable polymer layer were developed for the detection of biomolecules, which overcame the limitation of the Debye screening effect [78]. The authors constructed a permeable polymer layer by adsorption of pyrene butyric acid via π–π stacking, followed by covalent coupling of amine-terminated polyethylene glycol [79]. Curry and co-workers developed poly-L-lactide-based biodegradable piezoelectric force sensors to detect diaphragmatic contraction pressure. The device was fabricated by sandwiching poly-L-lactide layers between molybdenum electrodes and encapsulating them with polylactic acid (Figure 2). The authors found that the efficiency of the piezoelectric sensor depended on the crystallinity and degree of orientation of the polymer chains [80]. For sensitive detection of microRNAs, Salahuddin and co-workers [81] developed a *κ*-carrageenan-based mesoporous gold sensing system by dip coating *κ*-carrageenan hydrogel on the gold electrode for enhanced adsorption of microRNAs [82]. The authors found that the *κ*-carrageenan hydrogel network provides a 3D network for the binding of microRNAs, determined via chronocoulometry [81].

## 3. Quartz Crystal Microbalance (QCM)-Based Sensing Techniques

**Figure 3:**
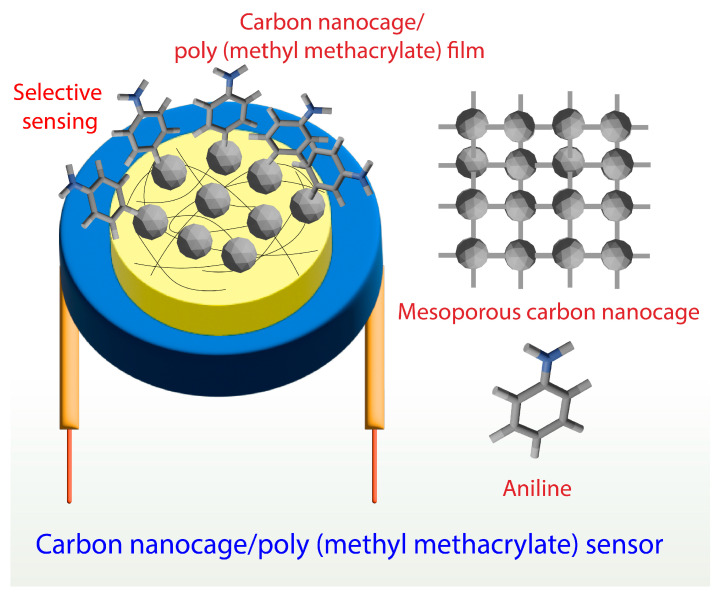
Highly sensitive mesoporous carbon nanocage-based sensor for the detection of aniline.

The detection of carcinogens, such as aromatic amines via noninvasive techniques, can be seen as a bright prospect in disease diagnostics [83,84]. A mesoporous carbon nanocage-based sensing system was developed to sense aromatic amines effectively. Mesoporous carbon nanocages with a larger surface area and pore volume were prepared using the cage-type mesoporous silica template technique [85]. The sensing system was developed by solvent-casting N, N-dimethylformamide, and poly (methyl methacrylate) films in combination with mesoporous carbon nanocages on a quartz crystal microbalance (QCM). The electrospun nanofibrous film containing carbon nanocages showed a larger surface area and porous membrane structure. Using a gas flow unit, the sensor was evaluated for its sensing capacity against aniline, benzene, toluene, ethanol, acetone, acetic acid, ammonia, and cyclohexane. The results showed that the developed carbon nanocage-based sensing system was more effective against aniline than benzene and toluene. We believe that hydrogen bonding with the amino group of aniline and active sites involving π−π forces could be the key to selective sensitivity [86,87]. Further investigation using density functional theory (DFT) also suggests that hydrogen bonding between the amine group in aniline and carbonyl group in carbon nanocage is necessary for high selectivity [88] (Figure 3).

Naturally derived carbon-based materials are being explored for their potential use in alcohol discrimination. We prepared a porous carbon material from naturally available grass and bamboo via chemical inactivation by phosphoric acid at 400 °C and tested its sensing capabilities. The prepared amorphous carbon materials were found to have hierarchical micro- and mesoporous structures with various oxygen-containing functional groups. We found that the porosity and surface area of the prepared carbon material can be controlled by regulating the impregnation ratio of phosphoric acid and bamboo. X-ray photoelectron spectroscopic (XPS) analysis revealed that hetero-carbon components with oxygen-containing functional groups are essential for sensing applications. The nanoporous bamboo carbon’s sensing capacity for volatile organic solvents was tested against various solvent vapors (methanol, ethanol, benzene, toluene, and acetic acid). The nanoporous carbon showed a higher sensitivity for non-aromatic solvent vapors (acetic acid, methanol, and ethanol) than aromatic solvent vapors (benzene and toluene). It was believed that the interaction between alcohol vapors and oxygen-containing surface functional groups such as –OH, C=O, and COOH on the nanoporous carbon, could be the reason for the sensitivity. The developed system also showed a significant sensitivity difference between methanol and ethanol for discriminating between C_1_ and C_2_ alcohols [89]. Hierarchical nanoporous fullerene structures are expected to display greater sensing capacity, owing to their unique π-conjugated structure, bonding interplay, and strong van der Waals reactions [90]. Fullerene and its derivatives have enhanced electronic, redox, and photonic properties, which can be used in sensing applications [91]. They show enhanced redox activity through their strong electron-accepting capacity. In this regard, Bitter melon-shaped nanoporous fullerene crystals were prepared and investigated for their vapor-sensing capacity. Powder diffraction analysis indicated both *fcc* and *hcp* phases, and after washing with isopropyl alcohol, the *hcp* phase disappeared. Raman scattering spectra indicated two A_g_ and six H_g_ bands. Particularly, the A_g_(2) band (pentagonal pinch mode) of the fullerene assembly is considered as the analytical probe, which showed that the free molecular rotation of the fullerene molecules is preserved in the self-assembled form. The prepared bitter melon-shaped fullerene crystals were subjected to vapors of water, methanol, hexane, benzene, toluene, and aniline. The system was able to sense aromatic solvent vapors such as benzene, toluene, and aniline, as evidenced from the significant frequency shift in QCM. These results suggest that the bitter melon-shaped fullerene crystals could serve more as a preferential host for aromatic vapors than other solvent vapors [92].

Continuing previous efforts, a tubular corn-husk-shaped fullerene assembly with a crystalline pore wall suitable for sensing applications was developed [93]. Corn-husk-shaped fullerene crystals were developed from pristine C_60_, using the dynamic liquid–liquid interface precipitation (LLIP) technique under ambient conditions. The fullerene crystals were tested for their sensing capacity against various solvent vapors, including methanol, ethanol, 2-propanol, formic acid, acetic acid, formaldehyde, acetone, pyridine, toluene, aniline, hexane, and cyclohexane. The fullerene crystals showed excellent sensitivity to acetic acid vapors by enhanced diffusion via mesoporous walls. The sensitivity towards acetic acid is mainly due to the reactivity of dimeric species from acetic acid in the vapor phase with electron-deficient fullerene molecules. Furthermore, the tubular wall with numerous microporous structures and crystalline pore walls helps to create nano gaps that are required for the adsorption of guest vapors. These results suggest that advanced sensing systems can be developed using fullerene rosette for the more effective sensing of formic acid [93]. In a recent report, Chen and coworkers prepared a micron-sized 2D fullerene rosette by self-assembling C_60_ with melamine/ethylenediamine and immobilized it onto a QCM resonator; a sensing system was developed. They found that the fullerene rosette exhibited an amorphous structure, unlike numerous fullerene assemblies generated by the LLIP technique. Raman scattering spectra indicated an A_g_(2) band shift which contributed to the restriction of free molecular rotation of the fullerene by strong interactions/covalent linking by melamine/ethylenediamine. Attenuated Total Reflection-Fourier Transform Infra-Red (ATR-FTIR) and XPS analysis also revealed the strong interaction of melamine/ethylenediamine components with fullerene molecules, making them suitable for sensing applications. The vapor-sensing property of the fullerene rosette was studied using various solvent vapors such as ethanol, ethyl acetate, formic acid, acetic acid, pyridine, acetone, hexane, aniline, benzene, toluene, and cyclohexane. The fullerene rosette showed better sensitivity against formic acid and acetic acid than other solvent vapors, mainly due to the available amino groups in the assembly [94].

**Figure 4:**
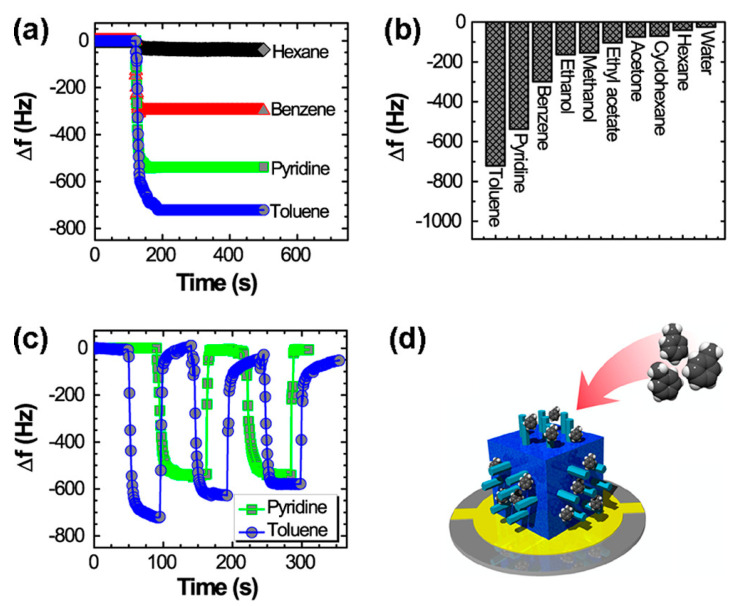
(**a**) QCM frequency shifts (Δ*f*) upon exposure to hexane, benzene, toluene, and pyridine. (**b**) Summary of sensing performance of hierarchically structured fullerene C_70_ cubes (HFC) (Z directed nanorods). (**c**) Typical repeatability tests up to a few cycles of an HFC electrode involving exposure and evacuation of pyridine and toluene vapors. (**d**) Schematic of HFC as a sensing antenna system. Reproduced from [97] with permission from the American Chemical Society, copyright 2016.

It is well known that the molecular sensing of mesoporous materials can be higher, owing to their larger surface areas [95]. However, fewer studies have reported the control over shape morphology with adjustments in organic solvent diffusion rates [96]. Bairi and co-workers developed a hierarchic fullerene C_70_ cube with mesoporous rods with crystalline pore walls, which functioned as the sensing antenna for toxic aromatic solvent vapors using ultrasound-assisted liquid−liquid interfacial precipitation [97]. Growth of the nanorods was controlled in the x, y, and z directions by modifying solvent diffusion conditions. Furthermore, the cubes with *z*-directed nanorods were tested for their vapor-sensing capacity using various solvent vapors (toluene, pyridine, hexane, cyclohexane, and benzene) via the QCM system (Figure 4).

We observed that adsorption of the aromatic solvent vapors (toluene and pyridine) was higher than that for aliphatic hydrocarbon vapors (hexane and cyclohexane). The difference in sensing capacity is believed to be the hallmark of grown nanorods on the surfaces of cubes. Strong π−π interactions with *sp*^2^ carbon-rich pore walls, mesoporous architecture, and very strong donor−acceptor charge-transfer interaction prove that fullerene-based structures can be widely used in sensing applications [98,99]. Structural modification is key to enhancing the vapor-sensing performance of fullerene assemblies. Modifying structures in a controlled manner requires several processes, including chemical etching, amination, electrochemical means, and so on [100,101]. Some researchers even increased the hydrophilicity of fullerenes to enhance the application of fullerene assemblies in various fields [102,103]. Surface modification strategies, including covalent and non-covalent adsorption with a range of polymerization techniques, are used to create active LbL films for sensing applications. These strategies offer control over the architecture for enhanced adsorption/recognition of the target molecules. We developed an LbL-based mesoporous carbon platform for the selective sensing of tea compounds. The mesoporous carbon surface was oxidized with the help of ammonium persulfate, and the LbL assembly was performed on a QCM surface using poly(diallyldimethyl chloride). The developed system effectively detected tannic acid via cooperative adsorption on the available nanoporous space [104].

**Figure 5:**
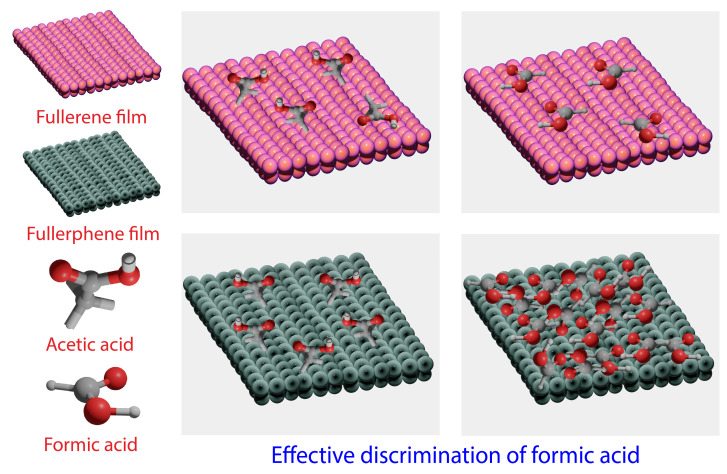
Atomic/molecular level molecular discrimination of formic acid by novel fullerphene films.

Our group successfully developed a strategy to efficiently control the morphology with face-selective etching on fullerene assemblies via chemical etching. Fullerene assemblies (1D—nanorods, 2D—nanosheets, and 3D—nanocubes) were prepared using the ultrasonic LLIP technique. By adding ethylene diamine (EDA) under gentle sonication, etching was performed to yield hollow tubular structures on the fullerene assemblies. After etching, all fullerene assemblies showed a broad N-H stretching vibration in infra-red spectra, indicating the presence of EDA-containing species. It was observed that the chemical etching did not affect the size and shape of the fullerene assemblies. The chemically etched fullerene nanostructures were tested for their sensing capabilities using various solvent vapors, including formic acid, acetic acid, benzene, and toluene. The hollow, porous nanostructures helped the aminated fullerene assemblies show excellent sensitivity for acid vapors (formic acid or acetic acid) over aromatic vapors (benzene or toluene) [105]. Possessing the sensitivity to differentiate one solvent from another is regarded as an excellent sensing material. A highly nanoporous novel material, fullerphene, was developed for its use in molecular discrimination at the atomic level. Nitrogen-doped fullerphene films were prepared using bottom-up fabrication with micro- and mesoporous structures. XPS analysis revealed that nitrogen exists as nitrogen pyrrolic-N and quaternary-N. It was found that nitrogen doping allows the fullerphene films to selectively absorb the low molecular-weight carboxylic acid vapors through noncovalent interactions. Enhanced surface area and porosity play an efficient role in the transport and diffusion of formic acid vapor over acetic acid (Figure 5). The spatially confined formic acid in the films experienced enhanced intermolecular interaction, resulting in atomic-level molecular discrimination [106].

## 4. Membrane-Type Surface Stress (MSS)-Based Sensing Platforms

**Figure 6:**
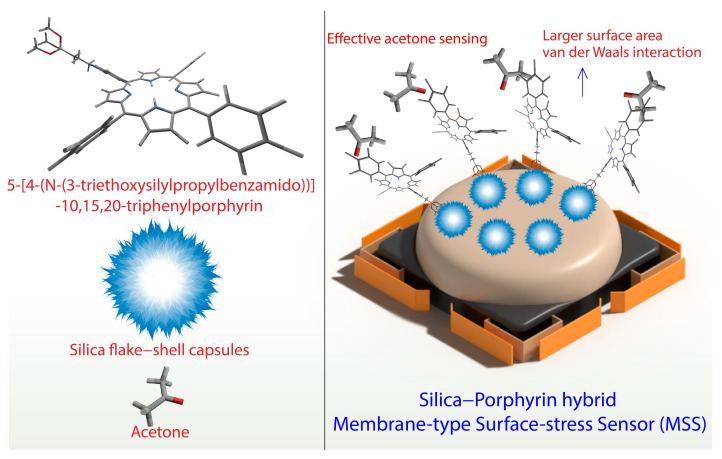
Silica–porphyrin hybrid assembly-based MSS system for effective detection of acetone.

Research on sensing alcohols has been studied using colorimetric discrimination and Raman-based techniques, among others. Membrane-Type Surface Stress sensors (MSS) display greater efficiency in sensing molecules in the gaseous phase and trace quantities [96]. Moreover, they are far more sensitive than the traditionally used piezoresistive cantilever sensors [107]. Several nanomechanical sensor systems with various ligands and functional groups have been attempted for the selective sensing of alcohols [108,109]. Recently, we developed a novel MSS-based sensor system coated with the copper complex, namely Cu (1,10-phenanthroline) ((±)-2,2′-bis(diphenylphosphino)-1,1′-binaphth-yl) hexafluorophosphate. The highly luminescent Cu (1,10-phenanthroline) ((±)-2,2′-bis(diphenylphosphino)-1,1′-binaphth-yl) hexafluorophosphate complex was prepared, and its chemical stability was analyzed using various spectroscopic studies. The MSS-based sensing system was prepared using direct inkjet spotting of the prepared complex over the membrane. The developed system showed excellent selectivity towards methanol vapor over a wide range of volatile organic compounds [110]. Hydrogen atoms from the aromatic C-H bonds and fluorine atoms from the hexafluorophosphate anions make weak interactions for the effective discrimination of methanol and related molecules [111]. Furthermore, the steric hindrance of the ligands forms a densely packed bulk layer that offers minimal intermolecular space for enhanced sensitivity of small molecules. The developed system exhibited clearer discrimination of methanol in *n*-hexane and gasoline than in ethanol mixtures [112]. We developed a novel hybrid inorganic-organic MSS system by conjugating silica flake−shell capsules with a tetraphenyl porphyrin derivative and its metal complex via covalent bonding. Silica flake−shell capsules were prepared using a self-template dissolution–regrowth mechanism to obtain a larger surface area and large silanol functional groups available for functionalization. Silica flake−shell capsules were functionalized with 5-[4-(N-(3-triethoxysilylpropylbenzamido))]-10,15,20-triphenylporphyrin and their metal complexes, including cobalt, nickel, copper, and zinc. Porphyrin derivatives are known for their excellent binding affinity towards inorganic molecules via various mechanisms, including van der Waals forces, π−π interactions, and coordination chemistry [113,114]. Porphyrins are a class of heterocyclic macrocycles that comprise four conjugated pyrrole rings arranged in a circle. Various hybrid assemblies were obtained, and their sensing properties were studied using MSS on model analytes such as acetone and nitric oxide. We found that the sensitivity of hybrid assemblies greatly relies on their interactions with acetone and nitric oxide via weak intermolecular forces (Figure 6).

**Figure 7:**
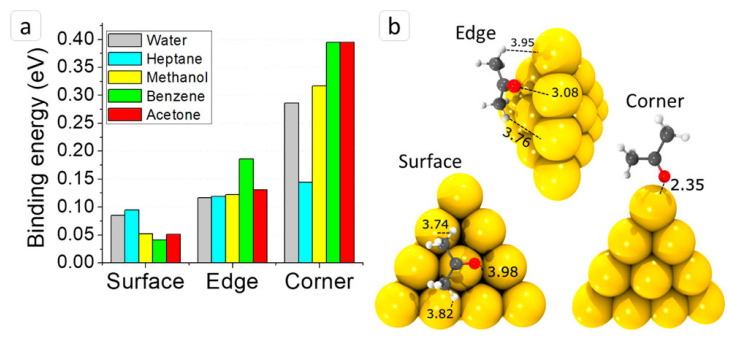
DFT calculation results: (**a**) binding energies of all analytes to the gold cluster Au_20_ at three sides, and (**b**) geometry of acetone molecule adsorption on Au_20_ surface, edge, and corner. Reproduced from [120] with permission from the American Chemical Society, copyright 2020.

The MSS-based hybrid inorganic-organic system exhibited excellent acetone sensing at ambient temperature conditions. The developed hybrid system showed a significant increase in the MSS response toward acetone vapors, due to the large surface area and available active sites. This can be seen as an excellent prospect in pharmaceutical industries that often rely on the production process where acetone is used under inert conditions [115]. In another study, we attempted to evaluate the sensing properties of gold nanocages deposited in MSS to detect volatile molecules. Gold nanocages were prepared using a galvanic replacement reaction with silver nanocubes as sacrificial templates after alloying and dealloying. Electron microscopic analysis revealed the presence of a hollow nanosized cubic structure with larger pores. The gold nanocages were deposited onto MSS via an inkjet-spotting technique to form a uniform layer and paired with a gas-sensing system. Furthermore, we evaluated the sensing capacity of gold nanocage MSS-based sensors against a wide range of solvents. During the adsorption of solvents, the measured signals reversibly returned to baseline, indicating that analytes briefly reacted with the gold nanocages via weak interactions, as evidenced during adsorption/desorption processes and DFT calculations [116,117]. The gold nanocage MSS-based sensing system showed maximum sensitivity to molecules with oxygen atoms in its structure. Hence, we believe the sensitivity could be due to intermolecular forces between O−Au (electron donor−acceptor bonding). DFT calculations also indicated the possibility of interactions between hydrogen atoms of the methyl group and gold [118,119]. The binding energy of the solvent vapor with the surface, edge, and corner of the gold nanocage was evaluated. It was shown that the energy is higher for solvent vapors, except for heptane, at the corner tip adsorption site (Figure 7). These results suggest that *sp*^2^/*sp*^3^-hybridized carbon creates electronic repulsion, which significantly influences the binding energy [120]. These nanostructures ultimately are used in the fabrication of sensing systems with effective functions. Some of the novel sensing platforms developed in recent years have been highlighted in Table 1.

## 5. Particle Systems

**Figure 8:**
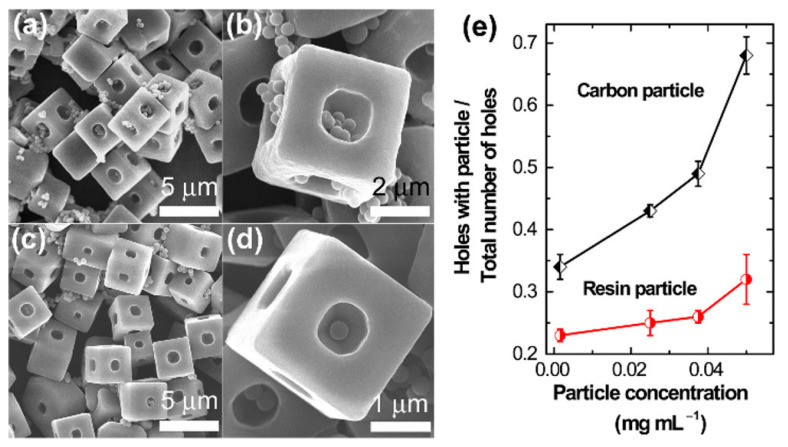
(**a**–**d**) SEM images showing different loading capacities of the OH-cubes toward carbon and polymer particles (0.05 mg/mL). (**e**) Plots of number of holes containing particle(s)/total number of holes vs. particle concentration. Reproduced from [121] with permission from the American Chemical Society, copyright 2017.

Manipulation of microscale structures is challenging, especially at the atomic and molecular level, for sensing applications [123]. Our group has applied several such strategies with fullerene self-assemblies to offer specific morphology and characteristics [124,125]. Continuing our previous efforts, we developed C_70_ fullerene cubes with a single open hole on each side, using the dynamic LLIP technique. We found that the growth of the cube follows a two-step process, as a solid core is formed immediately after adding C_70_ solution into *tert*-butyl alcohol, which functions as the nucleus for the crystal’s growth in the cubes. Powder diffraction analysis indicated the simple cubic structure of the prepared cubes as that of pristine C_70_, which possesses a hexagonal close-packed structure. The cubes had a solid core in the interior that can be controlled, as excess addition of C_70_ closes the hole, and a local electron beam irradiation ion beam can open the hole (Figure 8) [121].

**Figure 9:**
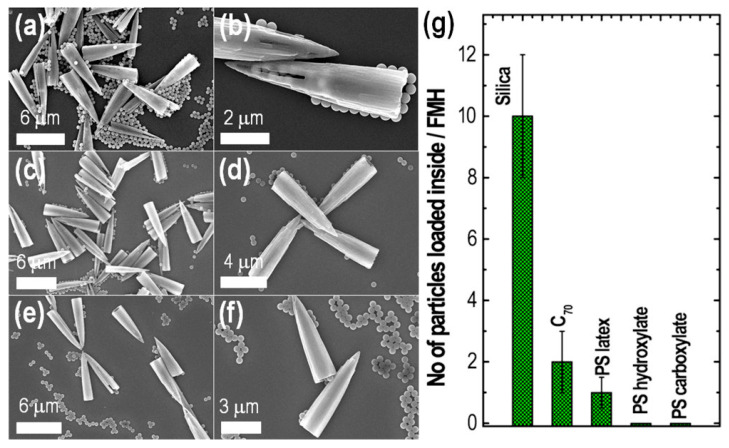
SEM observations showing the different loading capacities of fullerene microhorns (FMHs) toward (**a**,**b**) silica particles, (**c**,**d**) polystyrene (PS) latex particles, and (**e**,**f**) PS carboxylate particles. (**g**) Comparison of the different particles loaded inside the hollow space per FMH. Reproduced from [122] with permission from the American Chemical Society, copyright 2019.

Recognition of nano/microparticles such as graphitic and resin particles was attempted to check the preferential recognition properties. Scanning electron micrographs revealed that the holes in cubes were packed with graphitic particles compared to the resin particles of similar dimensions. The interaction of graphitic molecules with the cubes by strong π−π interaction between 3D *sp*^2^ carbon-rich open holes denote supramolecular interactions. Tang and coworkers have demonstrated the structural transformation of the fullerene microtube to the novel fullerene microhorn structure, which showed unusual microscopic particle recognition properties. Fullerene microtubes with a solid core at the center were produced by rapidly adding *tert*-butyl alcohol into a mixture of C_60_ and C_70_ solutions in mesitylene. The fullerene microtube, when treated with a solvent mixture of mesitylene and *tert*-butyl alcohol (3:1), resulted in the formation of novel fullerene microhorns. Electron microscopic analysis confirmed the presence of open hollow ends on the tubelike structures of fullerene microtubes. In contrast, fullerene microhorns were found to have a conical shape with a sharp solid tip and hollow tubular end. We found that structural transformation does not affect crystallinity. Polarized optical microscopic analysis suggests that fullerene microtubes break at the center to yield microhorns after applying a solvent mixture. Six H_g_ modes and two A_g_ modes Raman scattering spectra indicate the persistence of the free rotation of fullerene molecules in the microhorns. We performed the selective discrimination of the microparticles such as silica, polystyrene carboxylate, polystyrene hydroxylate, polystyrene latex, and C_70_ particles. Among them, the recognition of silica particles and their loading was higher than other particles, including C_70_ particles, which is anticipated to be more recognized considering its supramolecular π−π interactions. Owing to the net positive charge, preferential recognition was observed between microhorns and silica particles through strong electrostatic interactions [122] (Figure 9).

Similarly, Takashima and co-workers developed a 1,4,5,8-naphthalenediimide-based porous coordination polymer system, which can structurally transform to discriminate VOCs with exceptional sensitivity. They observed that the system could read aromatic molecules and signal them as photoluminescence in the visible light region via charge transfer or the heavy atom effect [126]. Some of the sensing platforms which utilize nanoarchitectonics have been developed as products, such as Breath Biopsy^®^ from Owlstone Medical (Cambridge, UK), exogenous VOC (EVOC^®^) probes from Owlstone Medical (Cambridge, UK), Cyranose^®^ 320 from Sensigent (Irwindale, CA, USA), etc., are available in the market. These examples indicate the necessity to integrate the nanoarchitectonics concept to achieve high sensitivity and selectivity in sensing industries ranging from chemical, biomedical, clinical, and other industrial settings.

## 6. Conclusions and Future Prospects

Advances in nanoscale-sensing systems concerning precision and efficacy continue to improve, thanks to advanced research in nanosensors. This review showcases the recent advancements of nanoarchitectonics-based sensing systems, where nanoscale architectures play an essential role in various medical, industrial, and chemical fields. Utilizing enhanced properties of nanoscale materials via nanoarchitectonics, including shape and size, will help better recognize target molecules. Nanoarchitectonics could be essential for fabricating well-defined nanostructures, including self-assembly, supramolecular chemistry, interfacial chemistry, chemical etching, and structural post-modifications, with a certain degree of control for precise discrimination of molecules. We understand that solvent vapors’ atomic/molecular level discrimination via sensing material depends on various chemical reactions/interactions such as electrostatic, host–guest, van der Waals forces, π−π interactions, and so on. Therefore, the ability to detect specific molecules with high sensitivity and spatial resolution via functionalization techniques involving covalent/non-covalent chemistries to achieve high-level selectivity is vital. This creates a desire to focus future research on tuning nanoscale structures for molecular recognition, and ultimately for sensing performance. Some promising materials, including fullerenes, gold nanostructures, DNA aptamers, and porphyrin-based composites, are bright prospects for developing novel yet effective sensing systems. Additionally, utilizing the interaction of light with metal nanostructures for developing nanoplasmonic sensing platforms are some of the novel concepts that are applying the idea of nanoarchitectonics. This understanding greatly improves the need to develop nanoscale sensors with atomic/molecular level discrimination, with advancements that forecast the potential of nanoarchitectonics in nanodevices for in vivo subjects with real-life monitoring of biological, industrial, and domestic environments.

## Figures and Tables

**Table 1:** List of recently developed sensors using nanoarchitectonics.

S. No	Sensing Platform	Detection	Sample	Reference
1	Nanometer-flat DNA thin film	QCM	Methanol	[54]
2	DNA nanotetrahedron	Electrochemical	Liver cancer cells	[55]
3	Carbon nanohorn	Electrochemical	Microcystin-leucine arginine	[57]
4	Cucurbit[6]uril-based hierarchic assembly	Fluorescence	Nitroaromatic compounds	[61]
5	Human angiotensin-converting enzyme 2 protein functionalized silver nanotriangle array sensor	Immobilization	Coronavirus	[71]
6	Boronate-Affinity Magnetic Immunity SERS Sensor	SERS	Alpha-Fetoprotein	[73]
7	Poly(methyl methacrylate) containing carbon nanocage sensor	QCM	Aniline	[88]
8	Bamboo-derived nanoporous carbon	QCM	VOCs	[89]
9	Bitter melon-shaped nanoporous C_60_ fullerene crystals	QCM	VOCs	[92]
10	Corn-Husk-Shaped C_60_ fullerene crystals	QCM	VOCs	[93]
11	C_60_ fullerene Rosette	QCM	VOCs	[94]
12	Hierarchical C_70_ fullerene Cube	QCM	VOCs	[97]
13	Fullerphene nanosheets	QCM	VOCs	[106]
14	Cu (1,10-phenanthroline) ((±)-2,2′-bis(diphenylphosphino)-1,1′-binaphth-yl) hexafluorophosphate	MSS	VOCs	[112]
15	Silica flake−shell capsules functionalized with 5-[4-(N-(3-triethoxysilylpropylbenzamido))]-10,15,20-triphenylporphyrin	MSS	VOCs	[115]
16	Au Nanocages	MSS	VOCs	[120]
17	C_70_ fullerene “Hole-in-Cube”	Particle	Carbon/resin particles	[121]
18	Fullerene microhorns	Particle	Silica, polystyrene and C_70_ particles	[122]

## Data Availability

Not applicable.

## References

[1] Liu J., Zhou H., Yang W., Ariga K. (2020). Soft Nanoarchitectonics for Enantioselective Biosensing. Acc. Chem. Res..

[2] Ariga K., Makita T., Ito M., Mori T., Watanabe S., Takeya J. (2019). Review of Advanced Sensor Devices Employing Nanoarchitectonics Concepts. Beilstein J. Nanotechnol..

[3] Takeuchi Y., Ohkura K., Nishina Y. (2023). Self-Assembly Strategies for Graphene Oxide/Silica Nanostructures: Synthesis and Structural Analysis. Bull. Chem. Soc. Jpn..

[4] Miyabe K., Aoki K. (2022). Moment Analysis of Solute Permeation Kinetics at an Interface of Mixed Micelles of Anionic and Nonionic Surfactants. Bull. Chem. Soc. Jpn..

[5] Nishiuchi T., Takeuchi S., Makihara Y., Kimura R., Saito S., Sato H., Kubo T. (2022). Synthesis, Properties, and Intermolecular Interactions in the Solid States of π-Congested X-Shaped 1,2,4,5-Tetra(9-Anthryl)Benzenes. Bull. Chem. Soc. Jpn..

[6] Fujita Y., Niizeki T., Fukumitsu N., Ariga K., Yamauchi Y., Malgras V., Kaneti Y.V., Liu C.-H., Hatano K., Suematsu H. (2021). Mechanisms Responsible for Adsorption of Molybdate Ions on Alumina for the Production of Medical Radioisotopes. Bull. Chem. Soc. Jpn..

[7] Segawa Y. (2022). Nonplanar Aromatic Hydrocarbons: Design and Synthesis of Highly Strained Structures. Bull. Chem. Soc. Jpn..

[8] Suzuki S., Homma A., Nishi R., Mizuno H., Kawauchi S., Fukuhara G. (2022). A Dynamically Responsive Chemosensor That Can Be Modulated by an Effector: Amplification Sensing by Positive Heterotropic Allosterism. Bull. Chem. Soc. Jpn..

[9] Watanabe H., Ekuni K., Okuda Y., Nakayama R., Kawano K., Iwanaga T., Yamaguchi A., Kiyomura T., Miyake H., Yamagami M. (2022). Composite Formation of Anthrylene- and Ferrocenoyl-Substituted Phenyleneethynylenes with Single-Wall Carbon Nanotubes (SWCNTs). Bull. Chem. Soc. Jpn..

[10] Miura C., Sanada Y., Katsumoto Y., Watanabe K. (2022). The Phase Behavior of a Mixture of the Ionic Liquids [C_8_mim][AzoO] and [C_8_mim][PF_6_]. Bull. Chem. Soc. Jpn..

[11] Lee G., Kageyama Y., Takeda S. (2022). Site-Selective Spin-Probe with a Photocleavable Macrocyclic Linker for Measuring the Dynamics of Water Surrounding a Liposomal Assembly. Bull. Chem. Soc. Jpn..

[12] Ariga K. (2021). Nanoarchitectonics for Analytical Science at Interfaces and with Supramolecular Nanostructures. Anal. Sci..

[13] Negi S., Hamori M., Kubo Y., Kitagishi H., Kano K. (2023). Monolayer Formation and Chiral Recognition of Binaphthyl Amphiphiles at the Air–water Interface. Bull. Chem. Soc. Jpn..

[14] Tanks J., Hiroi T., Tamura K., Naito K. (2023). Tethering Organic Disulfides to Layered Silicates: A Versatile Strategy for Photo-Controllable Dynamic Chemistry and Functionalization. Bull. Chem. Soc. Jpn..

[15] Mizuno K., Mori K., Matsuura S., Hashimoto T., Ishihara A. (2022). Selective Formation of P-Xylene in Catalytic Cracking of Low-Density Polyethylene Using Simultaneously Generated ZSM-5 and Mesoporous Silica with Gel Skeletal Reinforcement. Chem. Lett..

[16] Charles-Blin Y., Kondo T., Wu Y., Bandow S., Awaga K. (2022). Salt-Assisted Pyrolysis of Covalent Organic Framework for Controlled Active Nitrogen Functionalities for Oxygen Reduction Reaction. Bull. Chem. Soc. Jpn..

[17] Ariga K. (2018). Nanoarchitectonics approach for sensing. Materials Nanoarchitectonics.

[18] Arima H., Nakazono T., Wada T. (2022). Proton Relay Effects on Oxygen Reduction Reaction Catalyzed by Dinuclear Cobalt Polypyridyl Complexes with OH Groups on Bipyridine Ligands. Bull. Chem. Soc. Jpn..

[19] Psarra E., König U., Ueda Y., Bellmann C., Janke A., Bittrich E., Eichhorn K.-J., Uhlmann P. (2015). Nanostructured Biointerfaces: Nanoarchitectonics of Thermoresponsive Polymer Brushes Impact Protein Adsorption and Cell Adhesion. ACS Appl. Mater. Interfaces.

[20] Li C., Feng H., Xu H., Chen B., Yang T. (2022). An Intelligent Superhydrophilic/Underwater Superoleophobic Temperature Sensitive Switch Device with Excellent Targeted Oil-Water Separation Performance. Bull. Chem. Soc. Jpn..

[21] (Baitong) Tirayaphanitchkul C., (Jaa) Imwiset K., Ogawa M. (2020). Nanoarchitectonics through Organic Modification of Oxide Based Layered Materials; Concepts, Methods and Functions. Bull. Chem. Soc. Jpn..

[22] Arunbalaji S., Ismail M.A.M., Arivanandhan M., Alsalme A., Alghamdi A., Jayavel R. (2020). High Sensitive Electrochemical Nitrite Sensor Using Fe_2_O_3_/MoS_2_ Nanocomposites Synthesized by Facile Method. Bull. Chem. Soc. Jpn..

[23] Arnold G., Winkler R., Stermitz M., Orthacker A., Noh J.-H., Fowlkes J.D., Kothleitner G., Huth M., Rack P.D., Plank H. (2018). Tunable 3D Nanoresonators for Gas-Sensing Applications. Adv. Funct. Mater..

[24] Adachi J., Naito M., Sugiura S., Le N.H.-T., Nishimura S., Huang S., Suzuki S., Kawamorita S., Komiya N., Hill J.P. (2022). Coordination Amphiphile: Design of Planar-Coordinated Platinum Complexes for Monolayer Formation at an Air-Water Interface Based on Ligand Characteristics and Molecular Topology. Bull. Chem. Soc. Jpn..

[25] Kimura K., Yasunaga T., Makikawa T., Takahashi D., Toshima K. (2022). Efficient Strategy for the Preparation of Chemical Probes of Biologically Active Glycosides Using a Boron-Mediated Aglycon Delivery (BMAD) Method. Bull. Chem. Soc. Jpn..

[26] Van Tran V., Jeong G., Kim K.S., Kim J., Jung H.-R., Park B., Park J.-J., Chang M. (2022). Facile Strategy for Modulating the Nanoporous Structure of Ultrathin π-Conjugated Polymer Films for High-Performance Gas Sensors. ACS Sens..

[27] Veselinovic J., Almashtoub S., Nagella S., Seker E. (2020). Interplay of Effective Surface Area, Mass Transport, and Electrochemical Features in Nanoporous Nucleic Acid Sensors. Anal. Chem..

[28] Komiyama M., Yoshimoto K., Sisido M., Ariga K. (2017). Chemistry Can Make Strict and Fuzzy Controls for Bio-Systems: DNA Nanoarchitectonics and Cell-Macromolecular Nanoarchitectonics. Bull. Chem. Soc. Jpn..

[29] Liang X., Chen H., Li L., An R., Komiyama M. (2021). Ring-Structured DNA and RNA as Key Players In Vivo and In Vitro. Bull. Chem. Soc. Jpn..

[30] Nishijima Y., Juodkazis S. (2022). The Tunable Coupling between Metasurface and Molecular Vibration towards the Platform of Spectral Analysis. Bull. Chem. Soc. Jpn..

[31] Kawasaki Y., Nakagawa M., Ito T., Imura Y., Wang K.-H., Kawai T. (2022). Chiral Transcription from Chiral Au Nanowires to Self-Assembled Monolayers of Achiral Azobenzene Derivatives. Bull. Chem. Soc. Jpn..

[32] Mitomo H., Ijiro K. (2021). Controlled Nanostructures Fabricated by the Self-Assembly of Gold Nanoparticles via Simple Surface Modifications. Bull. Chem. Soc. Jpn..

[33] Rastogi R., Dogbe Foli E.A., Vincent R., Adam P.-M., Krishnamoorthy S. (2021). Engineering Electromagnetic Hot-Spots in Nanoparticle Cluster Arrays on Reflective Substrates for Highly Sensitive Detection of (Bio)Molecular Analytes. ACS Appl. Mater. Interfaces.

[34] Larasati L., Lestari W.W., Firdaus M. (2022). Dual-Action Pt(IV) Prodrugs and Targeted Delivery in Metal-Organic Frameworks: Overcoming Cisplatin Resistance and Improving Anticancer Activity. Bull. Chem. Soc. Jpn..

[35] Ohyoshi T., Zhao Y., Akemoto K., Ishihara T., Taniguchi A., Zhang M., Kigoshi H. (2022). Bioinspired Total Synthesis and Structure-Activity Relationship Studies on Aplaminal. Bull. Chem. Soc. Jpn..

[36] Hu K., Sun W., Tang R., Zhang B., An R., Liang X. (2022). Ethanolamine Derivatives Prompt Oxidation-Mediated Cleavage of Phosphorothioated DNA via Redox Control and Competition with Desulphurization. Bull. Chem. Soc. Jpn..

[37] Neal E.A., Nakanishi T. (2021). Alkyl-Fullerene Materials of Tunable Morphology and Function. Bull. Chem. Soc. Jpn..

[38] Nishikawa M., Kang H.G., Zou Y., Takeuchi H., Matsuno N., Suzuki M., Komatsu N. (2021). Conjugation of Phenylboronic Acid Moiety through Multistep Organic Transformations on Nanodiamond Surface for an Anticancer Nanodrug for Boron Neutron Capture Therapy. Bull. Chem. Soc. Jpn..

[39] Shrestha R.G., Maji S., Mallick A.K., Jha A., Shrestha R.M., Rajbhandari R., Hill J.P., Ariga K., Shrestha L.K. (2022). Hierarchically Porous Carbon from Phoenix Dactylifera Seed for High-Performance Supercapacitor Applications. Bull. Chem. Soc. Jpn..

[40] Kaneko M., Nakayama T., Seki H., Yamamoto S., Uemura T., Inoue K., Hadano S., Watanabe S., Niko Y. (2022). Lipophilic Nitrile N-Oxide for Catalyst-Free Surface Modification of Nanoemulsions as Light-Harvesting Nanoantennas. Bull. Chem. Soc. Jpn..

[41] Zeng R., Xu J., Liang T., Li M., Tang D. (2023). Photocurrent-Polarity-Switching Photoelectrochemical Biosensor for Switching Spatial Distance Electroactive Tags. ACS Sens..

[42] Yoshida K., Hayashi T., Takinoue M., Onoe H. (2022). Repeatable Detection of Ag^+^ Ions Using a DNA Aptamer-Linked Hydrogel Biochemical Sensor Integrated with Microfluidic Heating System. Sci. Rep..

[43] Chung S., Gurudatt N.G., Jeon J., Ban C., Shim Y.B. (2021). Fast Aptamer Generation Method Based on the Electrodynamic Microfluidic Channel and Evaluation of Aptamer Sensor Performance. Anal. Chem..

[44] Podder A., Lee H.J., Kim B.H. (2020). Fluorescent Nucleic Acid Systems for Biosensors. Bull. Chem. Soc. Jpn..

[45] Hiratsuka K., Salim F.T., Takahashi K., Nakamura T., Sagara Y. (2022). Crystal Structure of a 4,7-Bis(Phenylethynyl)-2,1,3-Benzothiadiazole-Based Cyclophane and the Mechanoresponsive Luminescence. Bull. Chem. Soc. Jpn..

[46] Ariga K., Yamauchi Y. (2020). Nanoarchitectonics from Atom to Life. Chem. Asian J..

[47] Liang X., Liu M., Komiyama M. (2021). Recognition of Target Site in Various Forms of DNA and RNA by Peptide Nucleic Acid (PNA): From Fundamentals to Practical Applications. Bull. Chem. Soc. Jpn..

[48] Itoh T., Procházka M., Dong Z.-C., Ji W., Yamamoto Y.S., Zhang Y., Ozaki Y. (2023). Toward a New Era of SERS and TERS at the Nanometer Scale: From Fundamentals to Innovative Applications. Chem. Rev..

[49] Karthick V., Shrestha L.K., Kumar V.G., Pranjali P., Kumar D., Pal A., Ariga K. (2022). Nanoarchitectonics Horizons: Materials for Life Sciences. Nanoscale.

[50] Fan S., Takada T., Maruyama A., Fujitsuka M., Kawai K. (2022). Large Heterogeneity Observed in Single Molecule Measurements of Intramolecular Electron Transfer Rates through DNA. Bull. Chem. Soc. Jpn..

[51] Imai Y., Mimura Y., Motomura Y., Ikemura R., Shizuma M., Kitamatsu M. (2023). Controlling Excimer-Origin Circularly Polarized Luminescence of Bipyrenyl-Arginine Peptides by Cyclodextrin in Water. Bull. Chem. Soc. Jpn..

[52] Utomo D.H., Kita M. (2023). Binding Mode of Actin–aplyronine A–tubulin Heterotrimeric Complex Revealed by Molecular Dynamics Simulation. Bull. Chem. Soc. Jpn..

[53] Liu M., Cui Y., Zhang Y., An R., Li L., Park S., Sugiyama H., Liang X. (2022). Single Base-Modification Reports and Locates Z-DNA Conformation on a Z-B-Chimera Formed by Topological Constraint. Bull. Chem. Soc. Jpn..

[54] Murata T., Minami K., Yamazaki T., Sato T., Koinuma H., Ariga K., Matsuki N. (2023). Nanometer-Flat DNA-Featured Thin Films Prepared via Laser Molecular Beam Deposition under High-Vacuum for Selective Methanol Sensing. Bull. Chem. Soc. Jpn..

[55] Sun D., Lu J., Luo Z., Zhang L., Liu P., Chen Z. (2018). Competitive Electrochemical Platform for Ultrasensitive Cytosensing of Liver Cancer Cells by Using Nanotetrahedra Structure with Rolling Circle Amplification. Biosens. Bioelectron..

[56] Pang P., Lai Y., Zhang Y., Wang H., Conlan X.A., Barrow C.J., Yang W. (2020). Recent Advancement of Biosensor Technology for the Detection of Microcystin-LR. Bull. Chem. Soc. Jpn..

[57] Zhang J., Lei J., Xu C., Ding L., Ju H. (2010). Carbon Nanohorn Sensitized Electrochemical Immunosensor for Rapid Detection of Microcystin-LR. Anal. Chem..

[58] Pang P., Teng X., Chen M., Zhang Y., Wang H., Yang C., Yang W., Barrow C.J. (2018). Ultrasensitive Enzyme-Free Electrochemical Immunosensor for Microcystin-LR Using Molybdenum Disulfide/Gold Nanoclusters Nanocomposites as Platform and Au@Pt Core-Shell Nanoparticles as Signal Enhancer. Sens. Actuators B Chem..

[59] Prakash R., Usha G., Karpagalakshmi K., Ramalakshmi S., Piramuthu L., Yang C., Selvapalam N. (2019). Vitamin B1 Sensor at Neutral PH and Improvement by Cucurbit[7]Uril. Bull. Chem. Soc. Jpn..

[60] Kim W., Hwang W., Kim N.H., Kim J., Baek K., Kim K. (2021). Permselective Two-Dimensional Polymer Film-Based Chemical Sensors. Bull. Chem. Soc. Jpn..

[61] Han X., Wang S., Liu M., Liu L. (2022). A Cucurbit[6]Uril-Based Supramolecular Assembly as a Multifunctional Material for the Detection and Removal of Organic Explosives and Antibiotics. Bull. Chem. Soc. Jpn..

[62] Harding D.P., Bootsma A.N., Wheeler S.E. (2019). Better Sensing through Stacking: The Role of Non-Covalent Interactions in Guanine-Binding Sensors. J. Phys. Chem. B.

[63] Chen X., Zhang Y., Arora P., Guan X. (2021). Nanopore Stochastic Sensing Based on Non-Covalent Interactions. Anal. Chem..

[64] Xiang D., Zhang Z., Han Z., Zhang X., Zhou Z., Zhang J., Luo X., Wang P., Zhao C., Li Y. (2020). Effects of Non-Covalent Interactions on the Properties of 3D Printed Flexible Piezoresistive Strain Sensors of Conductive Polymer Composites. Compos. Interfaces.

[65] Jimbo A., Nishikado Y., Imura K. (2021). Optical Field and Chemical Environment Near the Surface Modified Gold Nanoparticle Assembly Revealed by Two-Photon Induced Photoluminescence and Surface Enhanced Raman Scattering. Bull. Chem. Soc. Jpn..

[66] Wang X., Tian X., Zhao K., Wu L., Cao J., Shen S. (2022). Oxygen-Independent Free Radicals Induced by Photothermal Effect of Fe_3_O_4_ for Hypoxic Cancer Therapy. Chem. Lett..

[67] Jin C., Wu Z., Molinski J.H., Zhou J., Ren Y., Zhang J.X.J. (2022). Plasmonic Nanosensors for Point-of-Care Biomarker Detection. Mater. Today Bio.

[68] Mitomo H., Takeuchi C., Sugiyama R., Tamada K., Ijiro K. (2022). Thermo-Responsive Silver Nanocube Assembled Films. Bull. Chem. Soc. Jpn..

[69] Luo X., Zhu C., Saito M., Espulgar W.V., Dou X., Terada Y., Obara A., Uchiyama S., Tamiya E. (2020). Cauliflower-Like Nanostructured Localized Surface Plasmon Resonance Biosensor Chip for Cytokine Detection. Bull. Chem. Soc. Jpn..

[70] Chang C.-Y., Lin H.-T., Lai M.-S., Shieh T.-Y., Peng C.-C., Shih M.-H., Tung Y.-C. (2018). Flexible Localized Surface Plasmon Resonance Sensor with Metal–Insulator–Metal Nanodisks on PDMS Substrate. Sci. Rep..

[71] Yang Y., Murray J., Haverstick J., Tripp R.A., Zhao Y. (2022). Silver Nanotriangle Array Based LSPR Sensor for Rapid Coronavirus Detection. Sens. Actuators B Chem..

[72] Qiu G., Ng S.P., Liang X., Ding N., Chen X., Wu C.-M.L. (2017). Label-Free LSPR Detection of Trace Lead(II) Ions in Drinking Water by Synthetic Poly(mPD-*co*-ASA) Nanoparticles on Gold Nanoislands. Anal. Chem..

[73] He Y., Li L., Li X., Lin C., Zhang Y. (2021). Construction of Boronate-Affinity Magnetic Immunity SERS Sensor and Detection of Alpha-Fetoprotein (AFP) in Human Serum. Bull. Chem. Soc. Jpn..

[74] Sunayama H., Takamiya K., Takano E., Horikawa R., Kitayama Y., Takeuchi T. (2021). Simultaneous Detection of Two Tumor Marker Proteins Using Dual-Colored Signaling Molecularly Imprinted Polymers Prepared via Multi-Step Post-Imprinting Modifications. Bull. Chem. Soc. Jpn..

[75] Amishiro S., Ueda M., Mazaki Y. (2022). Synthesis, Structures, and Properties of Tropone-Fused Coumarin Dyes. Bull. Chem. Soc. Jpn..

[76] Ma J., He W., Meng F., Fu Y. (2022). 2-Methylimidazole-Induced Synthesis of 2D Amorphous FeCoNi Ternary Hydroxides Nanosheets by Mechanochemical Approach for Oxygen Evolution Reaction. Bull. Chem. Soc. Jpn..

[77] Heidari A., Mansouri-Torshizi H., Saeidifar M., Abdi K. (2021). Experimental and Computational Studies on the Interaction between DNA and BSA with a Couple of Isomeric [Pd(Daf)(Leu)]^+^, and [Pd(Daf)(Ile)]^+^ Antitumor Complexes, Their Synthesis and Spectral Characterization. Bull. Chem. Soc. Jpn..

[78] Yashima E., Maeda K. (2021). Helical Polymers with Dynamic and Static Macromolecular Helicity Memory: The Power of Helicity Memory for Helical Polymer Synthesis and Applications. Bull. Chem. Soc. Jpn..

[79] Gao N., Gao T., Yang X., Dai X., Zhou W., Zhang A., Lieber C.M. (2016). Specific Detection of Biomolecules in Physiological Solutions Using Graphene Transistor Biosensors. Proc. Natl. Acad. Sci. USA.

[80] Curry E.J., Ke K., Chorsi M.T., Wrobel K.S., Miller A.N., Patel A., Kim I., Feng J., Yue L., Wu Q. (2018). Biodegradable Piezoelectric Force Sensor. Proc. Natl. Acad. Sci. USA.

[81] Salahuddin B., Masud M.K., Aziz S., Liu C.-H., Amiralian N., Ashok A., Hossain S.M.A., Park H., Wahab M.A., Amin M.A. (2022). κ-Carrageenan Gel Modified Mesoporous Gold Chronocoulometric Sensor for Ultrasensitive Detection of MicroRNA. Bull. Chem. Soc. Jpn..

[82] Hamashita Y., Kise N., Sakurai T. (2021). Suppression of Intracellular Gene Expression by Inchworm-Type PNA-PEG Conjugate Depends on Recognition of a Monobasic Mutation. Bull. Chem. Soc. Jpn..

[83] Huo W., Miki K., Tokunaga D., Mu H., Oe M., Harada H., Ohe K. (2021). Dual-Stimuli-Responsive Probes for Detection of Ovarian Cancer Cells and Quantification of Both PH and Enzyme Activity. Bull. Chem. Soc. Jpn..

[84] Kumar V. (2020). Urea/Thiourea Based Optical Sensors for Toxic Analytes: A Convenient Path for Detection of First Nerve Agent (Tabun). Bull. Chem. Soc. Jpn..

[85] López-Salas N., Antonietti M. (2021). Carbonaceous Materials: The Beauty of Simplicity. Bull. Chem. Soc. Jpn..

[86] Hieda M., Tsujimura K., Kinoshita M., Matsumori N. (2022). Formation of a Tight Complex between Amphidinol 3 and Sterols in Lipid Bilayers Revealed by Short-Range Energy Transfer. Bull. Chem. Soc. Jpn..

[87] Yamamoto C., Suzuki M., Yoshida S. (2022). Pyridazine Synthesis from 1,2,4,5-Tetrazines and Alkynes in 1,1,1,3,3,3-Hexafluoro-2-Propanol through the Inverse Electron Demand Diels–Alder Reaction. Bull. Chem. Soc. Jpn..

[88] Kosaki Y., Izawa H., Ishihara S., Kawakami K., Sumita M., Tateyama Y., Ji Q., Krishnan V., Hishita S., Yamauchi Y. (2013). Nanoporous Carbon Sensor with Cage-in-Fiber Structure: Highly Selective Aniline Adsorbent toward Cancer Risk Management. ACS Appl. Mater. Interfaces.

[89] Shrestha L.K., Adhikari L., Shrestha R.G., Adhikari M.P., Adhikari R., Hill J.P., Pradhananga R.R., Ariga K. (2016). Nanoporous Carbon Materials with Enhanced Supercapacitance Performance and Non-Aromatic Chemical Sensing with C_1_/C_2_ Alcohol Discrimination. Sci. Technol. Adv. Mater..

[90] Maji S., Shrestha L.K., Ariga K. (2021). Nanoarchitectonics for Hierarchical Fullerene Nanomaterials. Nanomaterials.

[91] Matsumoto F., Sumino S., Iwai T., Ito T. (2021). Design of Linearly Substituted Fullerene Bis-Adducts with High Dielectric Constants Based on Theoretical Calculations. Bull. Chem. Soc. Jpn..

[92] Furuuchi N., Shrestha R.G., Yamashita Y., Hirao T., Ariga K., Shrestha L.K. (2019). Self-Assembled Fullerene Crystals as Excellent Aromatic Vapor Sensors. Sensors.

[93] Wei Z., Song J., Ma R., Ariga K., Shrestha L.K. (2022). Self-Assembled Corn-Husk-Shaped Fullerene Crystals as Excellent Acid Vapor Sensors. Chemosensors.

[94] Chen G., Bhadra B.N., Sutrisno L., Shrestha L.K., Ariga K. (2022). Fullerene Rosette: Two-Dimensional Interactive Nanoarchitectonics and Selective Vapor Sensing. Int. J. Mol. Sci..

[95] Baskar A.V., Ruban A.M., Davidraj J.M., Singh G., Al-Muhtaseb A.H., Lee J.M., Yi J., Vinu A. (2020). Single-Step Synthesis of 2D Mesoporous C_60_/Carbon Hybrids for Supercapacitor and Li-Ion Battery Applications. Bull. Chem. Soc. Jpn..

[96] Minami K., Imamura G., Tamura R., Shiba K., Yoshikawa G. (2022). Recent Advances in Nanomechanical Membrane-Type Surface Stress Sensors towards Artificial Olfaction. Biosensors.

[97] Bairi P., Minami K., Nakanishi W., Hill J.P., Ariga K., Shrestha L.K. (2016). Hierarchically Structured Fullerene C 70 Cube for Sensing Volatile Aromatic Solvent Vapors. ACS Nano.

[98] Maji S., Shrestha R.G., Lee J., Han S.A., Hill J.P., Kim J.H., Ariga K., Shrestha L.K. (2021). Macaroni Fullerene Crystals-Derived Mesoporous Carbon Tubes as a High Rate Performance Supercapacitor Electrode Material. Bull. Chem. Soc. Jpn..

[99] Islam M.S., Shudo Y., Hayami S. (2022). Energy Conversion and Storage in Fuel Cells and Super-Capacitors from Chemical Modifications of Carbon Allotropes: State-of-Art and Prospect. Bull. Chem. Soc. Jpn..

[100] Shan Y., Zhang G., Yin W., Pang H., Xu Q. (2021). Recent Progress in Prussian Blue/Prussian Blue Analogue-Derived Metallic Compounds. Bull. Chem. Soc. Jpn..

[101] Ivandini T.A., Einaga Y. (2021). Electrochemical Sensing Applications Using Diamond Microelectrodes. Bull. Chem. Soc. Jpn..

[102] Afreen S., Kokubo K., Muthoosamy K., Manickam S. (2017). Hydration or Hydroxylation: Direct Synthesis of Fullerenol from Pristine Fullerene [C_60_] via Acoustic Cavitation in the Presence of Hydrogen Peroxide. RSC Adv..

[103] Matsuo Y. (2021). Creation of Highly Efficient and Durable Organic and Perovskite Solar Cells Using Nanocarbon Materials. Bull. Chem. Soc. Jpn..

[104] Ariga K., Vinu A., Ji Q., Ohmori O., Hill J.P., Acharya S., Koike J., Shiratori S. (2008). A Layered Mesoporous Carbon Sensor Based on Nanopore-Filling Cooperative Adsorption in the Liquid Phase. Angew. Chem. Int. Ed..

[105] Hsieh C.-T., Hsu S., Maji S., Chahal M.K., Song J., Hill J.P., Ariga K., Shrestha L.K. (2020). Post-Assembly Dimension-Dependent Face-Selective Etching of Fullerene Crystals. Mater. Horizons.

[106] Song J., Murata T., Tsai K., Jia X., Sciortino F., Ma R., Yamauchi Y., Hill J.P., Shrestha L.K., Ariga K. (2022). Fullerphene Nanosheets: A Bottom-Up 2D Material for Single-Carbon-Atom-Level Molecular Discrimination. Adv. Mater. Interfaces.

[107] Shoji S., Stepanenko V., Würthner F., Tamiaki H. (2022). Self-Assembly of a Zinc Bacteriochlorophyll-d Analog with a Lipophilic Tertiary Amide Group in the 17-Substituent. Bull. Chem. Soc. Jpn..

[108] Shimizu T., Lungerich D., Stuckner J., Murayama M., Harano K., Nakamura E. (2020). Real-Time Video Imaging of Mechanical Motions of a Single Molecular Shuttle with Sub-Millisecond Sub-Angstrom Precision. Bull. Chem. Soc. Jpn..

[109] Shevchenko V.V., Gumenna M., Lee H., Klimenko N., Stryutsky O., Trachevsky V., Korolovych V., Tsukruk V. (2021). V Reactive Amphiphilic Aprotic Ionic Liquids Based on Functionalized Oligomeric Silsesquioxanes. Bull. Chem. Soc. Jpn..

[110] Shichijo K., Watanabe M., Hisaeda Y., Shimakoshi H. (2022). Development of Visible Light-Driven Hybrid Catalysts Composed of Earth Abundant Metal Ion Modified TiO2 and B12 Complex. Bull. Chem. Soc. Jpn..

[111] Kameda Y., Kowaguchi M., Amo Y., Usuki T., Okuyama D., Sato T.J. (2022). Experimental Determination of Deviation from Spherical Electron Densities of Atoms in Benzene Molecules in the Liquid State. Bull. Chem. Soc. Jpn..

[112] Nishikawa M., Murata T., Ishihara S., Shiba K., Shrestha L.K., Yoshikawa G., Minami K., Ariga K. (2021). Discrimination of Methanol from Ethanol in Gasoline Using a Membrane-Type Surface Stress Sensor Coated with Copper(I) Complex. Bull. Chem. Soc. Jpn..

[113] Hu X., Hu R., Wu X., Songsun F., Zhu H., Chen J., Chen H. (2021). Self-Assembled Fabrication of Water-Soluble Porphyrin Mediated Supramolecule-Gold Nanoparticle Networks and Their Application in Selective Sensing. Bull. Chem. Soc. Jpn..

[114] Miyaji A., Amao Y. (2022). Mechanism of Formate Dehydrogenase Catalyzed CO_2_ Reduction with the Cation Radical of a 2,2′-Bipyridinium Salt Based on a Theoretical Approach. Bull. Chem. Soc. Jpn..

[115] Osica I., Imamura G., Shiba K., Ji Q., Shrestha L.K., Hill J.P., Kurzydłowski K.J., Yoshikawa G., Ariga K. (2017). Highly Networked Capsular Silica-Porphyrin Hybrid Nanostructures as Efficient Materials for Acetone Vapor Sensing. ACS Appl. Mater. Interfaces.

[116] Singh B., Na J., Konarova M., Wakihara T., Yamauchi Y., Salomon C., Gawande M.B. (2020). Functional Mesoporous Silica Nanomaterials for Catalysis and Environmental Applications. Bull. Chem. Soc. Jpn..

[117] Zhang D., Liu D., Ubukata T., Seki T. (2021). Unconventional Approaches to Light-Promoted Dynamic Surface Morphing on Polymer Films. Bull. Chem. Soc. Jpn..

[118] Franchino A., Montesinos-Magraner M., Echavarren A.M. (2020). Silver-Free Catalysis with Gold(I) Chloride Complexes. Bull. Chem. Soc. Jpn..

[119] Itai T., Kuwamura N., Kojima T., Yoshinari N., Rujiwatra A., Konno T. (2021). Photoluminescent Ionic Solids of S-Bridged Gold(I)-Gallium(III) and Gold(I)-Indium(III) Hexanuclear Complexes. Bull. Chem. Soc. Jpn..

[120] Osica I., Melo A.F.A.A., Lima F.C.D.A., Shiba K., Imamura G., Crespilho F.N., Betlej J., Kurzydowski K.J., Yoshikawa G., Ariga K. (2020). Nanomechanical Recognition and Discrimination of Volatile Molecules by Au Nanocages Deposited on Membrane-Type Surface Stress Sensors. ACS Appl. Nano Mater..

[121] Bairi P., Minami K., Hill J.P., Ariga K., Shrestha L.K. (2017). Intentional Closing/Opening of “Hole-in-Cube” Fullerene Crystals with Microscopic Recognition Properties. ACS Nano.

[122] Tang Q., Maji S., Jiang B., Sun J., Zhao W., Hill J.P., Ariga K., Fuchs H., Ji Q., Shrestha L.K. (2019). Manipulating the Structural Transformation of Fullerene Microtubes to Fullerene Microhorns Having Microscopic Recognition Properties. ACS Nano.

[123] Sasaki Y., Lyu X., Tang W., Wu H., Minami T. (2021). Polythiophene-Based Chemical Sensors: Toward On-Site Supramolecular Analytical Devices. Bull. Chem. Soc. Jpn..

[124] Ariga K. (2022). Materials Nanoarchitectonics in a Two-Dimensional World within a Nanoscale Distance from the Liquid Phase. Nanoscale.

[125] Ariga K., Minami K., Shrestha L.K. (2016). Nanoarchitectonics for Carbon-Material-Based Sensors. Analyst.

[126] Takashima Y., Martínez V.M., Furukawa S., Kondo M., Shimomura S., Uehara H., Nakahama M., Sugimoto K., Kitagawa S. (2011). Molecular Decoding Using Luminescence from an Entangled Porous Framework. Nat. Commun..

